# Neurodegenerative processes accelerated by protein malnutrition and decelerated by essential amino acids in a tauopathy mouse model

**DOI:** 10.1126/sciadv.abd5046

**Published:** 2021-10-22

**Authors:** Hideaki Sato, Yuhei Takado, Sakiko Toyoda, Masako Tsukamoto-Yasui, Keiichiro Minatohara, Hiroyuki Takuwa, Takuya Urushihata, Manami Takahashi, Masafumi Shimojo, Maiko Ono, Jun Maeda, Asumi Orihara, Naruhiko Sahara, Ichio Aoki, Sachise Karakawa, Muneki Isokawa, Noriko Kawasaki, Mika Kawasaki, Satoko Ueno, Mayuka Kanda, Mai Nishimura, Katsuya Suzuki, Akira Mitsui, Kenji Nagao, Akihiko Kitamura, Makoto Higuchi

**Affiliations:** 1 Ajinomoto Co., Inc., Kawasaki 210-8681, Japan.; 2 Department of Functional Brain Imaging, National Institute of Radiological Sciences, National Institutes for Quantum and Radiological Sciences and Technology, Chiba 263-8555, Japan.; 3 Department of Cellular Neurobiology, Graduate School of Medicine, The University of Tokyo, Tokyo 113-0033, Japan.; 4 Department of Molecular Imaging and Theranostics, National Institute of Radiological Sciences, National Institutes for Quantum and Radiological Sciences and Technology, Chiba 263-8555, Japan.

## Abstract

Protein malnutrition is epidemiologically suggested as a potential risk factor for senile dementia, although molecular mechanisms linking dietary proteins and amino acids to neurodegeneration remain unknown. Here, we show that a low-protein diet resulted in down-regulated expression of synaptic components and a modest acceleration of brain atrophy in mice modeling neurodegenerative tauopathies. Notably, these abnormal phenotypes were robustly rescued by the administration of seven selected essential amino acids. The up-regulation of inflammation-associated gene expression and progressive brain atrophy in the tauopathy model were profoundly suppressed by treatment with these essential amino acids without modifications of tau depositions. Moreover, the levels of kynurenine, an initiator of a pathway inducing neuroinflammatory gliosis and neurotoxicity in the brain, were lowered by treatment through inhibition of kynurenine uptake in the brain. Our findings highlight the importance of specific amino acids as systemic mediators of brain homeostasis against neurodegenerative processes.

## Teaser

Intake of specific seven essential amino acids affects the pathology of the brain.

## INTRODUCTION

Recent studies in nutritional epidemiology have shown that protein intake is of vital importance for maintaining brain function in the elderly population (*1*, *2*). Protein ingestion in some elderly individuals, however, is insufficient (*3*–*6*). Oral issues such as decreased appetite with age (*7*, *8*), dysphagia (*9*), reduced muscle strength required for meat consumption (*10*, *11*), and periodontal disease (*12*) are noted as causes. Elderly people with dementia have lower protein intake than healthy elderly people (*13*, *14*). Low protein intake in elderly people raises the risk of mild cognitive impairment (*1*) and amyloid β accumulation in the brain (*2*). Moreover, a recent prospective cohort study demonstrated that lower blood levels of essential amino acids (EAAs), especially branched-chain amino acids, are associated with the development of Alzheimer’s disease (AD) (*15*), although the molecular mechanisms linking dietary proteins and amino acids to neurodegeneration remain unknown.

We recently demonstrated that a low-protein diet (LPD) resulted in cognitive dysfunctions in aged mice, such as learning disabilities, disinhibition, and hyperactive behavior, along with decreased amino acids and a consequent loss of neurotransmitters in the brain. The addition of seven EAAs—namely, Leu, Phe, and Lys supplemented with Ile, His, Val, and Trp, hereinafter referred to as “Amino LP7”—ameliorated these changes (*16*). In addition, in a clinical trial targeting adults 55 years or older, 12 weeks of ingestion of Amino LP7 resulted in improved attention and cognitive flexibility and psychosocial functioning (*17*), suggesting pivotal roles of EAAs in the homeostatic synthesis of neurotransmitters and brain functionality.

Expanding this indication, we further hypothesized that protein and amino acid intake may also modify pathological processes leading to neurodegenerative dementias, such as AD. In this study, we investigated the association between the intake of dietary proteins/Amino LP7 and elements of the etiological cascade, including fibrillary tau depositions, neuroinflammation, abnormal neurotransmission, synaptic impairment, and neuronal loss in rTg4510 mice, a mouse model of tauopathy (*18*, *19*).

## RESULTS

### Effect of protein and Amino LP7 intake on brain atrophy in rTg4510 mice

**Fig. 1.**
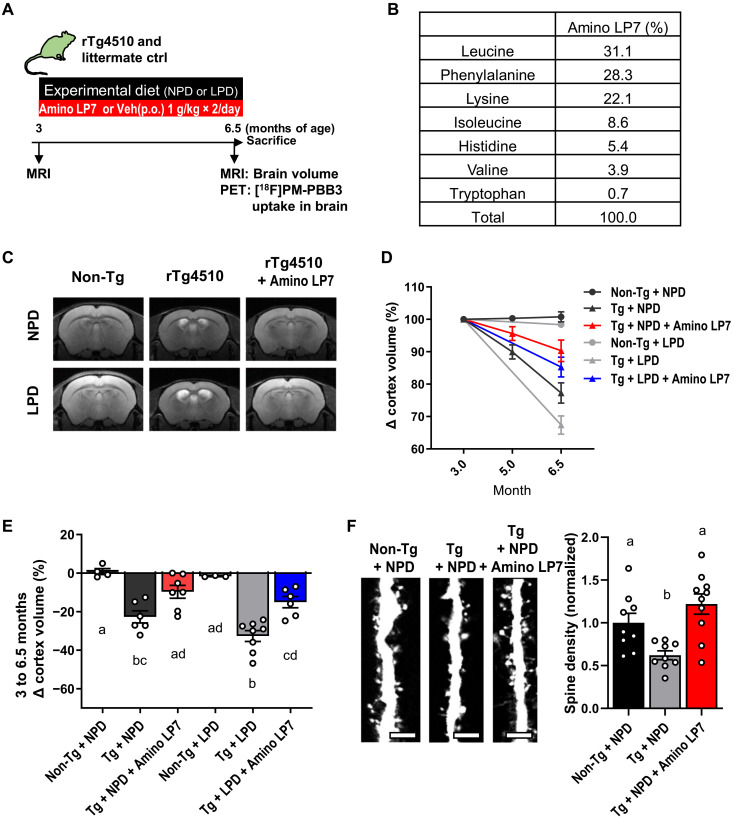
Neurodegenerative processes in rTg4510 mice are accelerated by an LPD and ameliorated by Amino LP7. (**A**) Schematic diagram of the experimental procedure. p.o., per os. (**B**) Composition of Amino LP7. (**C** to **E**) Representative coronal MR images (C), time course of cortex volume alterations (Δcortex volume) (D), and summary of Δcortex volume at 6.5 months (E) of mice in six conditions: rTg4510 mice or their littermate controls under NPD or LPD with or without Amino LP7 administration (*n* = 3 to 8 for each condition). Analysis of variance (ANOVA) [*F*_(5,28)_ = 16.98, *P* < 0.0001] with Tukey’s post hoc test was used. (**F**) Representative cortical spine images (left) and mean spine density (right) of control, rTg4510, and rTg4510 mice with Amino LP7 administration (*n* = 6 dendrites from three animals for each condition). Scale bars, 5 μm. ANOVA [*F*_(2,25)_ = 9.19, *P* = 0.001] with Tukey’s post hoc test was used. All data are expressed as the means ± SEM. Points represent individual animals. The difference between the means is not statistically significant (*P* ≥ 0.05) for all groups with the same alphabetical symbols and is statistically significant (*P* < 0.05) for two groups with different symbols. Non-Tg, littermate control; Tg, rTg4510.

First, we evaluated the effect of protein and Amino LP7 intake on brain cortical atrophy in rTg4510 mice. Because we used transgenic littermates tau/tetracycline-controlled transcriptional activator (tTA)(−/−) as controls, and the tTA gene is known to be associated with atrophy of the dentate gyrus (*20*), we focused on the cortex of rTg4510 mice to evaluate the treatment efficacy of Amino LP7 in all experiments. rTg4510 mice or their littermate controls were fed a normal protein diet (NPD) or LPD (table S1) with or without Amino LP7 administration from 3 to 6.5 months of age, and cortical volume and tau accumulation were assessed by magnetic resonance imaging (MRI) and positron emission tomography (PET) with a tau radioprobe [^18^F]PM-PBB3 (*21*) (Fig. 1A). Furthermore, we performed immunohistochemistry staining and Western blotting to understand the imaging results.

The Amino LP7 composition (Fig. 1B and table S2), which shows a high influx rate to the brain (*22*), was selected on the basis of its effectiveness in rescuing LPD-induced behavioral abnormalities (*16*). rTg4510 mice exhibited a greater than 20% decrease in cortical volume compared with control mice at 6.5 months, and intriguingly, the administration of Amino LP7 robustly mitigated this pathological change (Fig. 1, C to E). LPD accelerated atrophy by 10% in rTg4510 mice, although the effect was not statistically significant (Fig. 1, C to E). Although the cortical neuron area (NeuN-positive area) in rTg4510 mice was not significantly different than that of Amino LP7 administration under NPD, Amino LP7 administration could preserve the cortical neuron area under LPD (fig. S1).

Neither protein nor Amino LP7 intake significantly affected [^18^F]PM-PBB3-PET–detectable tau accumulation in the forebrains of rTg4510 mice at 6.5 months of age (fig. S2). In addition, Western blotting and immunostaining in relation to tau expression revealed no marked impacts on tau pathology of protein malnutrition and Amino LP7 intake (fig. S3). These findings indicate that the modulation of brain atrophy by dietary intervention was not secondary to the modification of tau pathologies but conceivably targeted downstream processes linking tau depositions and neuronal death in the etiological cascade.

The brain atrophy observed in rTg4510 mice was accompanied by a decrease in the cortical dendritic spine density, as assessed by postmortem microscopic measurements, which was also significantly ameliorated by Amino LP7 administration (Fig. 1F). The rTg4510 mice fed LPD showed a decreasing trend in spine density, which was partially recovered by Amino LP7 administration (fig. S4). This finding was more evident for C57BL/6J mice fed LPD (fig. S4). Histological data revealed that Amino LP7 rescued the decrease of homeostatic microglial marker P2Y12 receptor without significant change of Iba1-positive microglia in rTg4510 mice (fig. S1), suggesting a protective effect of Amino LP7 intake on microglial phenotypic conversion. In addition, the level of autophagosome formation, as an autophagy status, in rTg4510 mice was not affected by LPD or Amino LP7 (fig. S5).

### Effect of protein and Amino LP7 intake on brain gene expression in rTg4510 mice

**Fig. 2.**
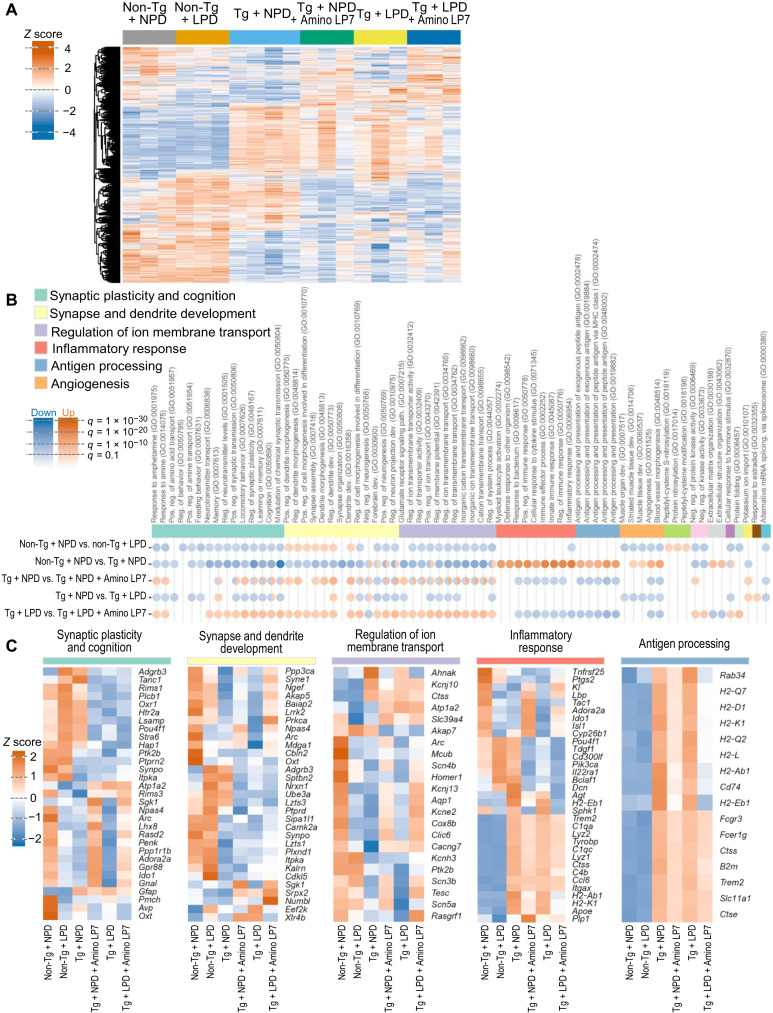
Alterations in brain gene expression in rTg4510 mice are accelerated by an LPD and ameliorated by Amino LP7. (**A**) Heatmap illustrating the relative expression of 7086 DEGs extracted from pairwise comparisons (non-Tg + NPD versus non-Tg + LPD; non-Tg + NPD versus Tg + NPD; Tg + NPD versus Tg + NPD + Amino LP7; Tg + NPD versus Tg + LPD; Tg + LPD versus Tg + LPD + Amino LP7). The vertical and horizontal axes indicate individual animals and DEGs, respectively. (**B**) Summary of GO enrichment analysis. GO terms enriched in down- or up-regulated DEGs were ranked according to *q* value, and the top 10 terms in each pairwise comparison were extracted. A circle indicates significant enrichment (*q* value < 0.05) of the GO term in the comparison, while its color and intensity indicate the direction (up/down) and *q* value of enrichment, respectively. GO terms are ordered and grouped according to automatically detected communities based on the similarity of comprising genes. (**C**) Heatmap illustrating the relative expression of representative genes in five categories. Vertical and horizontal axes indicate group mean and genes, respectively. The top 10 DEGs at the probe level contributing to significantly enriched GO terms in each pairwise comparison for each category were extracted and integrated. To improve visibility, probes indicating the same gene were summarized by the mean. Non-Tg, littermate control; Tg, rTg4510; pos., positive; neg., negative; reg., regulation; dev., development; path., pathway.

To gain insight into the mechanism underlying the effect of protein and Amino LP7 intake on brain atrophy, we performed global gene expression profiling of cortical samples derived from rTg4510 mice under NPD or LPD feeding with or without Amino LP7 supplementation and their littermate controls fed NPD or LPD. Five intergroup comparisons were conducted to investigate different diets (non-Tg + NPD versus non-Tg + LPD) and genotypes (non-Tg + NPD versus Tg + NPD) and the effects of diet (Tg + NPD versus Tg + LPD) and Amino LP7 (Tg + NPD versus Tg + NPD + Amino LP7 and Tg + LPD versus Tg + LPD + Amino LP7) in rTg4510 mice. The five comparisons yielded 1702, 4323, 1797, 1961, and 1710 differentially expressed genes (DEGs), respectively; a heatmap of the 7086 unique DEGs identified in the six groups is shown in Fig. 2A. The most noticeable difference was observed between rTg4510 and the control mice, which appeared to be mitigated by the administration of Amino LP7. To biologically interpret these differences, we performed gene ontology (GO) enrichment analysis on up- and down-regulated DEGs in each comparison. The top 10 GO terms in each comparison were extracted and are summarized in Fig. 2B. The effect of dietary intervention per se in control mice was centered around GO terms involved in neuronal function and structure (categorized as “synaptic plasticity and cognition,” “synapse and dendrite development,” and “regulation of ion membrane transport”), and this result does not contradict a previous report (*16*). Partial up-regulation in terms of GO related to neuronal development may indicate compensative changes to cover the loss of neurotransmitters in LPD-fed mice, although further exploration is needed. In rTg4510 mice, GO terms related to “inflammatory response” and “antigen processing” were robustly up-regulated, while GO terms involved in neuronal function and structure were down-regulated compared with control mice, in agreement with previous observations (*23*, *24*). LPD accelerated the alterations in the down-regulated GO terms without noticeably up-regulating neuroinflammatory and neuroimmune responses in rTg4510 mice, while Amino LP7 administration reversed both the up- and down-regulated GO terms in LPD- and NPD-fed animals. Moreover, the effect of Amino LP7 on the down-regulated GO terms was more prominent in rTg4510 mice fed LPD, implying a marked amelioration of synaptic integrity by Amino LP7 even under malnourishment conditions. Representative genes contributing to these categories and their relative expression levels are shown in Fig. 2C. Among these genes were those well known to be associated with synaptic activities such as *Homer1* and *Arc*, glial activation such as *Ccl6*, *C1q*, and *Gfap*, and genetic AD risks involving glial changes such as *Apoe* and *Trem2*. Inflammatory changes shown by *Tnfa* and *Cox1* and *Cox2* and glial activation shown by *Iba1* and *Cd11b* by quantitative polymerase chain reaction (qPCR) further supported the gene expression profiling of cortical samples (fig. S6). The gene expression data indicate that Amino LP7 may exert therapeutic efficacies on synapses not only by reversing amino acid deficiencies in LPD-fed rTg4510 mice but also by reinforcing the functionalities and organizations of neurons burdened with tau fibrils in NPD-fed animals.

To examine whether this synaptic rescue by Amino LP7 is mediated by modifications of neurotoxic tau species, we carried out additional experiments in rTg4510 mice and aged C57BL/6J mice without tau abnormalities. LPD decreased the neuronal firing rates measured by calcium signals in an intravital two-photon laser microscopic assay (figs. S7 and S8) and dendritic spine density according to histological measurements (fig. S4), which were reversed by Amino LP7 administration. Regarding the influence of LPD, these results are consistent with the behavioral deficits of aged C57BL/6J mice induced by LPD and their reversal by Amino LP7 (*16*).

Hence, Amino LP7 could protect synapses against toxic insults related and unrelated to tau abnormalities, presumably by the maintenance of synaptic activities through a sustained synthesis of key neurotransmitters, resulting in activity-dependent local mRNA translation of *Homer1* and *Arc* in spines (*25*). The expression levels of *Fgf14* variants were not altered by each food condition (fig. S9).

### Brain kynurenine elevation in rTg4510 mice is ameliorated by Amino LP7

**Fig. 3.**
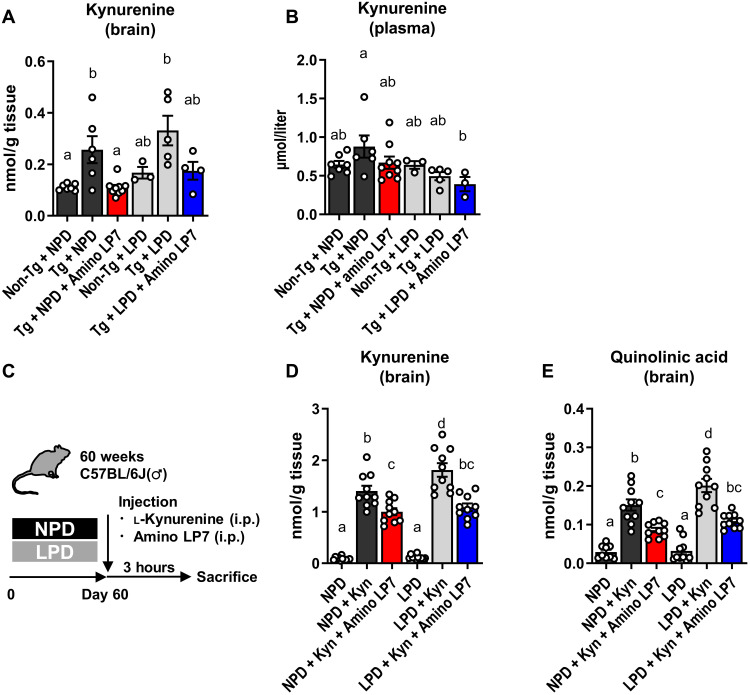
Brain kynurenine elevation in rTg4510 is ameliorated by Amino LP7. (**A** and **B**) Brain (A) and plasma (B) kynurenine concentrations (*n* = 3 to 9 for each condition). ANOVA [brain, *F*_(5,28)_ = 7.32, *P* = 0.0002; plasma, *F*_(5,27)_ = 2.66, *P* = 0.04] with Tukey’s post hoc test was used. (**C**) Schematic diagram of acute intraperitoneal injection of l-kynurenine with or without Amino LP7 in aged C57BL/6J mice under NPD or LPD. (**D** and **E**) Brain kynurenine (D) and QA (E) concentrations after acute l-kynurenine injections with or without Amino LP7 (*n* = 9 to 11 for each condition). ANOVA [kynurenine, *F*_(5,56)_ = 82.14, *P* < 0.0001; QA, *F*_(5,53)_ = 37.73, *P* < 0.0001] with Tukey’s post hoc test was used. All data are expressed as the means ± SEM. Points represent individual animals. The difference between the means is not statistically significant (*P* ≥ 0.05) for all groups with the same alphabetical symbols and is statistically significant (*P* < 0.05) for two groups with different symbols. Non-Tg, littermate control; Tg, rTg4510; Kyn, kynurenine.

Microarray profiling has also highlighted crucial roles played by Amino LP7 in the suppression of tau-induced neuroinflammatory processes. This effect may not be attributable to a simple reversal of amino acid insufficiencies because LPD alone did not exacerbate inflammation or immune activation. In light of the fact that EAAs with high rates of influx to the brain (*22*) via specific transporters [L-type amino acid transporter 1 (LAT-1)] were selected as primary constituents of Amino LP7, we hypothesized that Amino LP7 competitively inhibits the blood-to-brain transfer of toxic amino acid metabolites, such as kynurenine, which share the same transporters with Amino LP7 (*26*, *27*). Kynurenine in the central nervous system (CNS) is metabolized by glial cells, and its downstream metabolite, quinolinic acid (QA), is known to exert neurotoxic effects (*28*). In addition, proinflammatory, pro-oxidant, and excitotoxic properties of QA have been implicated in the pathogenesis of AD (*29*). rTg4510 mice fed NPD and LPD demonstrated higher kynurenine concentrations in the brain than controls, which was completely reversed by the administration of Amino LP7 (Fig. 3A). An elevation of plasma EAA concentrations by Amino LP7 administration was also observed, further supporting our hypothesis (table S3). Plasma kynurenine was not influenced by LPD or Amino LP7 (Fig. 3B), indicating that altered kynurenine levels in the brain were not consequent to modified systemic production and metabolism of kynurenine by dietary interventions. To directly confirm the competitive suppression of kynurenine influx into the brain by Amino LP7, we intraperitoneally injected saline, kynurenine, or kynurenine + Amino LP7 into aged C57BL/6J mice fed NPD or LPD and examined kynurenine and QA concentrations at 3 hours after administration (Fig. 3C). Kynurenine and QA levels in the brain were elevated by the administration of kynurenine, which was robustly suppressed by the simultaneous injection of Amino LP7 in both NPD and LPD conditions (Fig. 3, D and E).

These results indicate that Amino LP7 competitively suppresses the transporter-mediated blood-to-brain transfer of kynurenine, thereby ameliorating neurotoxic inflammation in rTg4510 mice. To examine whether kynurenine affects synaptic function and structure, we carried out additional experiments in aged C57BL/6J mice without tau abnormalities. Kynurenine decreased the neuronal firing rates measured by calcium signals in an intravital two-photon laser microscopic assay (fig. S8) and dendritic spine density according to histological measurements (fig. S4). It is speculated that, along with the supplementation of neurotransmitters, Amino LP7 may disrupt the vicious cycle of neurotoxic inflammation and neuronal deterioration, eventually counteracting neuronal death and consequent brain atrophy. As kynurenine is primarily metabolized by glial cells, the involvement of deleterious gliosis, which has been reported to be a primary accelerator of neuronal death in tauopathy models (*30*, *31*), may play a significant role in this therapeutic mechanism, although the details require further investigation.

## DISCUSSION

Here, we demonstrated the importance of protein and amino acid nutrition for maintaining brain homeostasis against neurodegenerative processes. Protein malnutrition led to perturbed synaptic integrity and functionality in wild-type (WT) and tauopathy model mice without pronounced induction of neuroinflammation or neuronal loss. Chronic intake of a specific EAA composition—Leu, Phe, and Lys supplemented with Ile, His, Val, and Trp, which was composed to directly match the ratios of the brain influx rate of the different EAAs (*22*)—rescued synapses from LPD-provoked insults and, more significantly, drastically reversed inflammatory responses and brain atrophy in the tauopathy model.

In this study, LPD contained ^1^/_4_ of the dietary protein content compared with NPD. Although LPD has more carbohydrate content than NPD, our previous report shows that blood glucose levels were not significantly different between the groups (*16*), and thus, carbohydrates are considered not to be relevant to these findings. Regarding spine density, protein deficiency alone had a significant impact, the density of which was reduced by nearly 40% because of LPD in non-Tg mice. Compared with Tg mice fed NPD, whose spine density was reduced by approximately 50%, LPD alone had a marked, comparable effect on spine loss in non-Tg mice. In non-Tg mice, LPD did not cause brain atrophy, and neuronal deficits did not seem to be prominent, indicating that LPD causes more synaptic disorders than neuronal deficits. Nutritional epidemiological studies suggest that protein ingestion in some elderly individuals is insufficient (*3*–*6*), suggesting a relationship between dietary protein deficiency and cognitive decline (*1*, *2*). Decreased appetite with age (*7*, *8*), dysphagia (*9*), reduced muscle strength required for meat consumption (*10*, *11*), and periodontal disease (*12*) are the major issues for reduced protein consumption in elderly individuals. To the best of our knowledge, the spine density of elderly people with insufficient protein intake has not been investigated thus far, but analogous to this result, there is a possibility that spine density in the brain has decreased in these populations as well. When discussing relationships between food intake and the brain, there are recent reports indicating that caloric restriction (CR) has positive effects on longevity and neurodegenerative diseases in animal studies (*32*–*34*). Müller *et al.* (*34*) indicated that CR attenuated β amyloid deposition and increased autophagy in an AD model. In our experiments, caloric intake did not differ much in any group, and our data showed that autophagy in rTg4510 mice was not affected under either food condition (fig. S5). The differences with previous reports, especially about CR, may be due to the effects of differences in total calorie intake or the effects of differences in the intake of individual nutrients such as proteins and amino acids or the duration of intake. Thus, further studies are needed to determine how protein and amino acid insufficiency specifically contribute to the reduction of spine density and, if any, how it is related to the pathology of dementia, such as amyloid β accumulation in the brain (*2*).

One of the intriguing findings in this study is the brain kynurenine elevation in rTg4510 mice fed NPD. Kynurenine in the CNS is metabolized by glial cells, and its downstream metabolite, QA, is known to exert neurotoxic effects (*28*). In addition, proinflammatory, pro-oxidant, and excitotoxic properties of QA have been implicated in the pathogenesis of AD (*29*). The reason why kynurenine in the brain increased in rTg4510 mice is currently unknown. It is possible that uptake into the brain increased with the activation of astrocytes and microglia in the brain, but further studies are needed to support this hypothesis. Because the kynurenine elevation was reversed at least by peripheral EAA administration, it is considered that the uptake of kynurenine into the brain, not the production in the brain, is possibly related to kynurenine elevation. This might be one mechanism that explains the etiology of rTg4510 mice; further research on this is necessary. Notably, although kynurenine was not increased in WT mouse brains with LPD, LPD seemed to cause synaptic disorders. Because some gene expression levels (*Maob*, *Iba1*, and *Cd11b*) of astrocytes and microglia were increased in WT mouse brains with LPD (fig. S6) while kynurenine was not elevated, there should be other unknown effects of LPD on the brain in addition to kynurenine elevation.

We hypothesized that the rate of amino acid influx to the brain (*22*) would reflect the requirement of each amino acid to maintain brain homeostasis against neurodegenerative processes and composed the EAA mixture rich in Leu, Phe, and Lys to directly match the ratios of the brain influx rate of the different EAAs. The beneficial effect of this EAA mixture, Amino LP7, was not directly associated with pathological tau fibril formation. Together with the results of our prior study (*16*), it is presumable that Amino LP7 acts on multiple mechanisms, including neurotransmitter compensation and competitive inhibition of kynurenine influx into the brain, providing therapeutics against diverse neurodegenerative conditions involving reciprocal links between neuronal deterioration and neuroinflammation. Most neurotransmitters are synthesized from amino acids. For example, dopamine and norepinephrine are synthesized from Tyr, which is a metabolite of Phe. Glu is synthesized from Leu, and 30 to 50% of the amino groups of Glu and Gln in the brain are derived from Leu (*35*). Amino LP7 is mainly composed of Leu, Phe, and Lys, which are potential substrates for synthesizing Glu in brain cells (*35*). Glu is known to be an important neurotransmitter that triggers de novo spine growth (*36*) and is involved in learning and memory ability (*37*). As the constituents of Amino LP7 have high rates of influx to the brain (*22*) via specific transporters (e.g., LAT-1), it is plausible that Amino LP7 not only delivered the neurotransmitter substrate into the brain but also competitively inhibited the blood-to-brain transfer of toxic amino acid metabolites, such as kynurenine, which share the same transporters (*26*, *27*). This may mimic the effects of dietary protein ingestion to maintain brain homeostasis against neurodegenerative processes. Because the composition of Amino LP7 was designed to match the amino acid influx to the brain, it includes Trp, whose downstream metabolite is kynurenine. Although the amount of Trp in Amino LP7 is quite low, it should be examined whether Amino LP7 without Trp could be more effective in future studies.

Tauopathy mouse models are pervasively characterized by tau-mediated neurodegeneration leading to brain atrophy, which can be readily detected by MRI (*38*). Neurodegeneration is induced by synapse loss, which is phagocytosed by microglia (*39*). Our study showed tau-mediated brain atrophy, synapse loss, and microgliosis, which were all ameliorated by Amino LP7 administration. Because no significant association between tau pathology and brain atrophy was observed here, causative factors need further investigation. Of them, the heterodeficiency of *Fgf14* caused by transgene integration may affect the progress of neuronal loss observed in rTg4510 mice (*40*). To clarify this possibility, we performed reverse transcription qPCR (RT-qPCR) to measure the mRNA level of each *Fgf14* splice variant (V1, V2, X1, and X2). Our data showed no significant difference in variant expression levels in NPD- and LPD-fed rTg4510 mice with or without Amino LP7 administration (fig. S9). Therefore, heterodeficiency of *Fgf14* may not be associated with protein malnutrition–induced atrophy.

The potency of nutritional intervention as simple as Amino LP7 to prevent the progression of functional, structural, and immunological pathogenesis at a nonclinical level is intriguing and may serve as a previously unidentified nutritional approach to dementia prevention. As Amino LP7 contains dietary nutrients, the current therapeutic paradigm is promptly applicable to humans, and a clinical trial for these components is now ongoing.

## MATERIALS AND METHODS

### Animals

Male C57BL/6J (55 to 63 weeks, Charles River Laboratories, Japan) and rTg4510 mice were used for experiments. The rTg4510 mouse line overexpressing human 4R0N tau with the P301L mutation was licensed from Mayo Clinic, bred, and maintained at Taconic, Hudson, NY. Briefly, the mice were generated by crossing the activator line (129S6 strain, Taconic) expressing tTA driven by the CaMKIIα promoter and the responder line (FVB/NCrl strain, Charles River Laboratories) expressing human 4R0N tau with the P301L mutation driven by tetracycline-operon responsive element, as described (*19*). Transgenic littermates tau/tTA(−/−) were used as controls. Mice were housed in their cages at 25°C under a 12-hour light/12-hour dark cycle (lights on at 8:00 p.m. for C57BL/6J mice and at 7:00 a.m. for rTg4510 mice). All animal experimental procedures in the present study were approved by the institutional review board of the animal ethical committee and followed the institutional guidelines of Ajinomoto Co., Inc. and the Institutional Committee for Animal Experimentation of the National Institutes for Quantum and Radiological Science and Technology.

### Diet and amino acid intervention

Mice were provided ad libitum access to water and a control diet (NPD; 20% casein-based; table S1). At the start of the experimental protocol, the control diet was replaced with the experimental diet, corresponding to either a normal protein (NPD; 20% casein-based) or low protein (LPD; 5% casein-based) diet (table S1). For intervention with amino acids, we treated mice per os with 0.5% methylcellulose (10 ml/kg; vehicle) or 1 g/10 ml/kg Amino LP7 (Fig. 1B) twice daily on days 1 to 5 of each week throughout the experimental period.

### MRI and PET imaging and image analysis

The MRI scan was conducted with a 7.0T horizontal MRI scanner (Bruker Biospin, Germany) with a volume coil for transmission (Bruker Biospin) and a two-channel phased-array surface coil (Rapid Biomedical, Germany) for reception. Mice were anesthetized with 1.5 to 2.0% isoflurane, and their rectal temperature was maintained at 36.5 ± 0.5°C with a heating pad and warm air during the experiment. T2-weighted MR images were obtained by rapid acquisition with a relaxation enhancement sequence and the following parameters: repetition time/effective echo time (TR/effective TE) = 4200/36 ms, number of averages = 8, rapid acquisition with a relaxation enhancement (RARE) factor = 8, number of slices = 28, matrix = 256 × 256, and scan time = 17 min, 55 s.

PET scans were performed using a microPET Focus 220 animal scanner (Siemens Medical Solutions, Germany), yielding 95 transaxial slices 2.0 mm (center-to-center) apart, a 19.0-cm transaxial field of view (FOV), and a 7.6-cm axial FOV. Before the scans, the mice were anesthetized with 1.5% (v/v) isoflurane. Emission scans were carried out for 60 min ([^18^F]PM-PBB3) in three-dimensional (3D) list mode with an energy window of 350 to 750 keV immediately after intravenous injection of [^18^F]PM-PBB3. All list-mode data were sorted into 3D sinograms, which were then Fourier-rebinned into 2D sinograms (frames 4 × 1, 8 × 2, and 14 × 5 min). Average images were generated with maximum a posteriori reconstruction, and dynamic images were reconstructed with filtered backprojection using a 0.5-mm Hanning’s filter.

All captured PET and MR images were coregistered and analyzed using PMOD software (PMOD Technologies, Japan). Volumes of interest (VOIs) were manually generated by anatomical alignment with the cortex, hippocampus, and cerebellum of MR images. Regional radioactivities were quantified by the standardized uptake value ratio with the cerebellum as a reference region at 40 to 60 min after [^18^F]PM-PBB3 injection. For measurement of regional brain volumes, the anatomical VOI was manually segmented on a T2-weighted MRI in each slice to define the neocortex using PMOD software.

### Plasmid construction and AAV preparation

Full-length complementary DNAs encoding GCaMP6s [a gift from D. Kim, Addgene plasmid no. 40753 (*41*)] were amplified by PCR and subcloned into the multicloning site of adeno-associated virus (AAV) transfer plasmid containing a rat synapsin promoter, a woodchuck posttranscriptional regulatory element, and a polyA signal flanked with inverted terminal repeats. For large-scale preparation of recombinant AAV, AAV plasmid and AAV serotype DJ packaging plasmids (pHelper and pRC-DJ) were introduced into human embryonic kidney–293T cells with polyethyleneimine transfection. Forty-eight hours after transfection, cells were harvested and lysed, and AAV particles were subsequently purified with a HiTrap heparin column (GE healthcare, UK) as described previously (*42*).

### Spine evaluation

For virus injection, mice were deeply anesthetized by inhalation of isoflurane mixed with air and placed in a stereotaxic instrument (Narishige, Japan). Holes were drilled in the skull above the target region. Then, 0.5 μl of virus solution was bilaterally injected into the neocortex [barrel field of the primary somatosensory cortex (S1BF); anterior-posterior −1.25 mm, medial-lateral ±3.0 mm, dorsal-ventral −0.5 mm from bregma]. The injected virus was prepared by mixing the two virus solutions: a stock solution of pAAV.CAG.LSL.tdTomato (a gift from H. Zeng, Addgene viral prep no. 100048-AAV1) and a diluted solution of pENN.AAV.CaMKII0.4.Cre. SV40 (a gift from J. M. Wilson, Addgene viral prep no. 105558-AAV1). Six weeks after virus injection, the mice were deeply anesthetized with an overdose of pentobarbital and then transcardially perfused with phosphate-buffered saline (PBS) followed by 4% paraformaldehyde in PBS (fixation buffer). Brain tissues were removed from perfused mice and postfixed overnight with fixation buffer, cryoprotected in 30% sucrose, and frozen in Optimal Cutting Temperature (OCT) compound (Sakura Finetek, Japan) with ice-cold isopentane. The brain tissues were sliced into 40-μm-thick serial sections using a cryostat, and the sections were collected in PBS followed by permeabilization with 0.3% Triton X-100/PBS for 5 min. The sections were incubated with a blocking solution (1% bovine serum albumin/5% normal goat serum/0.3% Triton X-100/PBS) for an hour at room temperature (25°C) and then incubated overnight at 4°C with mouse anti–red fluorescent protein antibody (MBL, Japan; 1:1000) diluted in blocking solution. After washing with 0.3% Triton X-100/PBS, tissue sections were incubated overnight at 4°C with Alexa Fluor–conjugated secondary antibodies (Thermo Fisher Scientific, USA; 1:1000) in blocking solution. After washing, the sections were incubated with Hoechst 33342 (2 μg/ml) and mounted on slides in tissue-mounting medium (Polyscience, USA). Immunofluorescence images were acquired using a laser scanning confocal microscope (Zeiss LSM880 with Airyscan, Germany) at ×40 magnification [numerical aperture of 1.2] objective. All stacks were obtained with a constant image-acquisition setting. For spine data analysis, dendrites expressing tdTomato in the neocortex were randomly selected from four mouse brains in each group. Dendritic spines were automatically detected by using our original pipeline in 3D data analysis software (arivis Vision4D, USA), and then the number of spines per unit length (spine density) and the volume of the detected spines were measured in the software. The measured spine density and spine volume were normalized by the average in the control group.

### Microarray gene expression analysis

Total RNA was isolated and purified from the neocortex of extracted brains as described previously. RNA concentration and purity were assessed by a NanoDrop spectrophotometer (Thermo Fisher Scientific, Waltham, MA, USA), and RNA integrity was analyzed on an Agilent Bioanalyzer (Santa Clara, CA, USA). Gene expression analysis was performed using a Single-color Agilent Microarray SurePrint G3 Mouse 8x60K (version 2.0; Agilent Technologies). Hybridization, washing, scanning, and quantification were performed according to the array manufacturer’s recommendations.

Raw intensity data were background-corrected using the normexp method and between-array normalized by the quantile method to yield the log_2_-transformed expression intensity. Only probes that were both (i) positive for the “gIsFound” flag in all samples and (ii) positive for “gIsWellAboveBG” in all samples in at least one group were used for further analysis. Replicate probes were collapsed by calculating the mean. Two technical replicates were analyzed for each sample, which were averaged to singularly represent each sample. DEGs between the two groups were identified by the weighted average difference (WAD) (*43*), which ranks the genes according to fold change weighted by mean expression. Probes with |WAD| > 0.1 criterion were extracted as DEGs and used in further analysis.

Data processing and analysis of gene expression were performed using R (version 3.4.3). The following Bioconductor packages were used: “limma” (version 3.34.9) for preprocessing, clusterProfiler (version 3.6.0) and “org.Mm.eg.db” (version 3.5.0) for GO enrichment analysis, and igraph (version 1.2.4.1) for detection of communities within gene ontologies.

### Two-photon imaging and image analysis

For the surgical procedure, the animals were anesthetized with a mixture of air, oxygen, and isoflurane (3 to 4% for induction and 2% for surgery) via a facemask, and a cranial window (3 to 4 mm in diameter) was attached over the left somatosensory cortex, centered at 1.8 mm caudal and 2.5 mm lateral from bregma. A custom metal plate was affixed to the skull with a 7-mm-diameter hole centered over the cranial window. The method for preparing the chronic cranial window has been previously reported elsewhere (*44*, *45*). All experiments were performed 2 weeks after cranial window surgery. The awake animal was placed on a custom-made apparatus, and real-time imaging was conducted by wide-field two-photon laser scanning microscopy (Multiphoton Mesoscope, Thorlabs, NJ, USA) with an excitation wavelength of 900 nm. An emission signal was detected with a bandpass filter for GCaMP (525/50 nm). The visual field size of the image was 2000 μm by 2000 μm, and the in-plane pixel size was 1 μm. The temporal resolution was 1 or 4 Hz for 60 s (60 to 240 frames per trial) depending on the size of the FOV. Images were acquired at a depth of approximately 150 μm from the brain surface.

In the experiment, 10 trials were successively performed with an intertrial interval of 60 s. Thirty seconds after the start of the scan, activation of neurons was induced by sensory stimulation with an air puff for 10 s. In addition, spontaneous neuronal activities were measured for 240 s using the same scan parameters as the stimulation experiment.

Two-photon imaging was performed in awake mice. The experimental protocol for measurements by two-photon imaging was as reported previously (*45*). Briefly, the metal plate on the animal’s head was screwed to a custom-made stereotactic apparatus. The animal was then placed on a Styrofoam ball that was floating using a stream of air. This allowed the animal to exercise freely on the ball while the animal’s head was fixed to the apparatus.

Image analysis of two-photon calcium imaging was performed using MATLAB (MathWorks, MA, USA). For analysis of sensory stimulation experiments, data on timing when the mouse moved were first removed. Next, motion correction was performed using NoRMCorre (*46*). The images for all time points were divided by the averaged image from 0 to 30 s at the resting state, and the images of the signal change rate were obtained. Regions of interest (ROIs) were manually drawn on the image obtained by maximum intensity projection processing for images of the sensory stimulation period. The time-dependent change in the average luminance value was then obtained for each ROI. In the analysis of spontaneous neuronal activity experiments, the time-dependent change in the average luminance value was obtained for each ROI following motion correction. For the correction of baseline fluctuations, the rate of signal changes for the baseline was calculated following the procedure of Jia *et al.* (*47*). Neural firing was defined as a relative fluorescence signal exceeding 0.5.

### Quantification of amino acid concentrations in the plasma

A previously described quantification method for amino acids (*48*) was used in this study with minor modifications. Briefly, plasma samples were mixed with the internal standard solution (stable isotope-labeled amino acids in water) and deproteinized with acetonitrile. The plasma samples were derivatized with APDSTAG (FUJIFILM Wako Pure Chemicals, Osaka, Japan) and analyzed using liquid chromatography coupled with tandem mass spectrometry (LC-MS/MS).

### Quantification of tryptophan metabolite concentrations in plasma and brain tissue

Tryptophan metabolites were analyzed using a previously described quantification method (*49*). Plasma samples were mixed with the internal standard solution (stable isotope-labeled tryptophan metabolites in water) and deproteinized with acetonitrile. The frozen brain tissue was powdered using a Multi-Beads Shocker and homogenized with methanol aqueous solution containing the internal standard solution and chloroform. The sample solution was subjected to LC-MS/MS.

### Kynurenine injection

Three hours before sacrifice, saline (vehicle) or 33 mg/10 ml/kg l-kynurenine was injected intraperitonially with methylcellulose (vehicle) or Amino LP7 in aged C57BL/6J mice that were fed an experimental diet for 60 days.

### Biochemical experiment

#### Tissue extraction

Brain extraction was performed according to what was previously reported (*50*). Briefly, mouse brains were bisected down the midline to yield two hemispheres after euthanizing by cervical dislocation. The cerebral cortex and hippocampus of the right hemisphere of each animal were quickly frozen on dry ice and stored at −80°C until use. Tissues were then homogenized in 10 volumes of tris-buffered saline [TBS; 50 mM tris/HCl (pH 7.4), 274 mM NaCl, 5 mM KCl, 1% protease inhibitor mixture (Sigma-Aldrich, St. Louis, MO), 1% phosphatase inhibitor cocktail I and II (Sigma-Aldrich), and 1 mM phenylmethylsulfonyl fluoride (PMSF)]. After a portion of homogenates (total homogenate) was saved, the remaining homogenates were centrifuged at 27,000*g* for 20 min at 4°C to obtain supernatant (S1) and pellet fractions. Pellets were homogenized in five volumes of high salt/sucrose buffer [0.8 M NaCl, 10% sucrose, 10 mM tris/HCl, (pH 7.4), 1 mM EGTA, and 1 mM PMSF] and centrifuged as described above. The supernatants were collected and incubated with sarkosyl (1% final concentration; SERVA Electrophoresis GmbH) for 1 hour at 37°C, followed by centrifugation at 150,000*g* for 1 hour at 4°C to obtain salt and sarkosyl-extractable (S3) and sarkosyl-insoluble (P3) fractions. The P3 pellet was resuspended in TE buffer [10 mM tris/HCl (pH 8.0) and 1 mM EDTA] to a volume equivalent to half of that of the brain specimens used to produce brain homogenates.

#### Western blotting and dot blotting

Total homogenates and fractionated tissue extracts were dissolved in SDS-sample buffer containing β-mercaptoethanol (2.5%). The heat-treated samples (55°C for 15 min) were separated by gel electrophoresis on 10% tris-glycine SDS–polyacrylamide gel electrophoresis gels (Nakarai, Japan) and transferred onto nitrocellulose membranes (Bio-Rad Laboratories, Hercules, CA). After blocking with a blocking solution containing 5% nonfat milk and 0.05% Tween 20 in TBS, the membranes were incubated with primary antibodies, such as Tau12 (provided by P. Davies, Feinstein Institutes for Medical Research), Tau46 (no. 4019, Cell Signaling, Danvers, MA), AT8 (MN1020, Thermo Fisher Scientific, Waltham, MA), LC3 (CAC-CTB-LC3-1-50, Cosmo Bio, Tokyo, Japan), and β-actin (A1987, Merck). After washing with TBS–Tween 20, the membranes were incubated with peroxidase-conjugated goat anti-rabbit antibodies (1:5000; Jackson ImmunoResearch, West Grove, PA) or anti-mouse immunoglobulin G (1:5000; Jackson ImmunoResearch). Bound antibodies were detected using an enhanced chemiluminescence system (ECL PLUS kit; PerkinElmer). Western blot immunoreactivity was visualized by Amersham Imager 600 (GE Healthcare). Quantitative analysis was performed with Image Studio Lite software (LI-COR, Lincoln, NE).

#### Histological examination

The animals were deeply anesthetized with sodium pentobarbital and transcardially perfused with saline, and brain tissues were removed. The brains of rTg4510 mice and non-Tg mice were fixed with 4% paraformaldehyde in phosphate buffer and cryoprotected using 20% sucrose in phosphate buffer. The brains were embedded and frozen in OCT compound (Sakura Finetek Japan, Tokyo, Japan), and 20-μm-thick frozen sections were generated in a cryostat (HM560; Thermo Fisher Scientific, Waltham, MA). The brain sections were immunostained with mouse monoclonal antibodies against tau phosphorylated at Ser^202^ and Thr^205^ (AT8, 1:250; Thermo Fisher Scientific, MN1020) and Iba1 (1:1000; Merck Millipore, MABN92), rabbit polyclonal antibodies against P2Y12 (1:10,000, raised in our laboratory) (*51*), and guinea pig polyclonal antibodies against NeuN (1:2500, Merck Millipore, ABN90). Immunolabeling was examined using DM4000 (Leica, Wetzlar, Germany) and BZ-X710 (Keyence, Osaka, Japan) microscopes. Images were analyzed using a BZ-X Analyzer (Keyence, Osaka, Japan).

### Reverse transcription quantitative polymerase chain reaction

Total RNA was extracted and purified from the frozen cortex using an RNeasy mini kit (Qiagen) following the manufacturer’s instructions. The primer sequences are shown in table S4. Single-strand complementary DNA was synthesized with 0.43 μg of extracted RNA using SuperScript IV VILO Master Mix (Thermo Fisher Scientific). RT-qPCR was performed with the 7900HT Fast Real-Time PCR System (Applied Biosystems). PCR was conducted as follows: denaturation for 60 s at 95°C followed by 45 cycles at 95°C for 15 s and 60°C for 60 s; for *fgf14*, PCR was conducted as follows: denaturation for 20 s at 95°C followed by 40 cycles at 95°C for 1 s and 60°C for 20 s. mRNA expression levels of each *fgf14* variant were quantified relative to a reference gene, *Hprt*, and other genes were quantified relative to a reference gene, *Gapdh*.

### Statistics

Statistical analyses were performed using GraphPad Prism 6 Software. Data were statistically analyzed by unpaired *t* test for two comparisons and one-way analysis of variance (ANOVA) with Tukey’s post hoc test for ≥3 comparisons. *P* values of <0.05 were considered statistically significant at a confidence interval of 95%.

## Notes

View/request a protocol for this paper from *Bio-protocol*.
