# Supplementary material for: Neurodegenerative processes accelerated by protein malnutrition and decelerated by essential amino acids in a tauopathy mouse model
Source: Sci Adv. 2021 Oct 22;7(43):eabd5046. doi: 10.1126/sciadv.abd5046 (PMC8535828; doi:10.1126/sciadv.abd5046)
Supplement: Supplementary file 1 — Figs. S1 to S9 Tables S1 to S4 [file sciadv.abd5046_sm.pdf]

Science Advances
AAAS

Supplementary Materials for

Neurodegenerative processes accelerated by protein malnutrition and decelerated by essential amino acids in a tauopathy mouse model

Hideaki Sato, Yuhei Takado, Sakiko Toyoda, Masako Tsukamoto-Yasui, Keiichiro Minatohara, Hiroyuki Takuwa, Takuya Urushihata, Manami Takahashi, Masafumi Shimojo, Maiko Ono, Jun Maeda, Asumi Orihara, Naruhiko Sahara, Ichio Aoki, Sachise Karakawa, Muneki Isokawa, Noriko Kawasaki, Mika Kawasaki, Satoko Ueno, Mayuka Kanda, Mai Nishimura, Katsuya Suzuki, Akira Mitsui, Kenji Nagao, Akihiko Kitamura*, Makoto Higuchi*

*Corresponding author. Email: higuchi.makoto@qst.go.jp (M.H.); akihiko.kitamura.7y9@asv.ajinomoto.com (A.K.)

Published 22 October 2021, *Sci. Adv.* **7**, eabd5046 (2021)
DOI: 10.1126/sciadv.abd5046

This PDF file includes:

Figs. S1 to S9
Tables S1 to S4

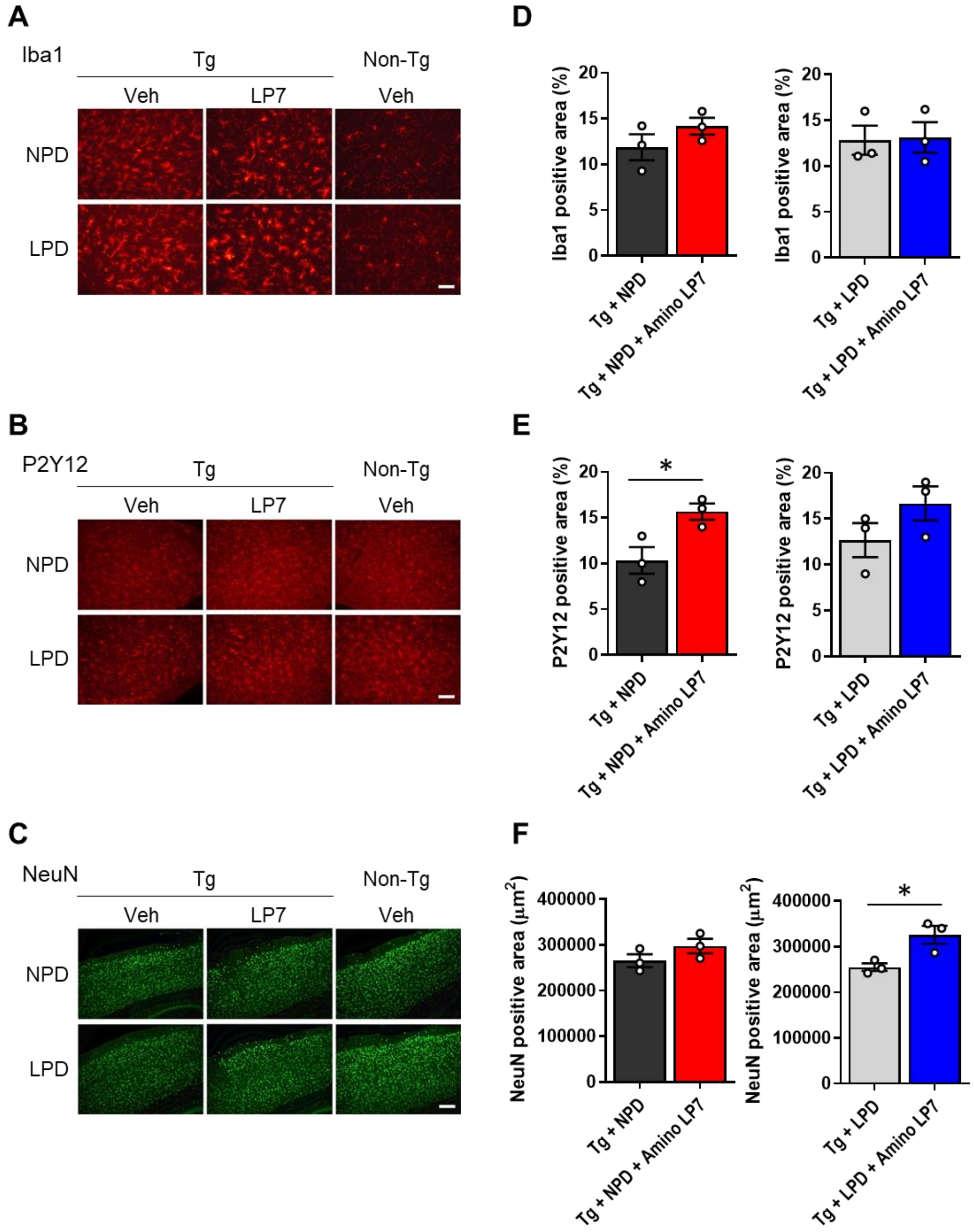

Fig. S1: Immunohistochemical analysis in the cortex of rTg4510 mice treated with Amino LP7 under a normal or a low protein diet.
(**A** to **C**) Immunostaining of cortical sections from 6.4- to 7.1-month-old rTg4510 mice (Tg) treated with vehicle (Veh) or Amino LP7 (LP7) under NPD or LPD and 6.3- to 6.4-month-old nontransgenic mice (Non-Tg) treated with vehicle under NPD or LPD with Iba1 (A), P2Y12 (B), and NeuN (C) antibodies. Scale bars, 50 μm (A), 100 μm (B), and 250 μm (C). (**D** to **F**) Quantitative analysis of the ratio of the Iba1-positive area (D) and the P2Y12-positive area (E), and the NeuN-positive area (F) in the cortex of 6.4- to 7.1-month-old rTg4510 mice treated with vehicle or Amino LP7 under NPD or LPD conditions (n = 3 for each condition). All data are expressed as the mean ± SEM. Points represent individual animals. *: $P < 0.05$, unpaired t-test. ns, not significant.

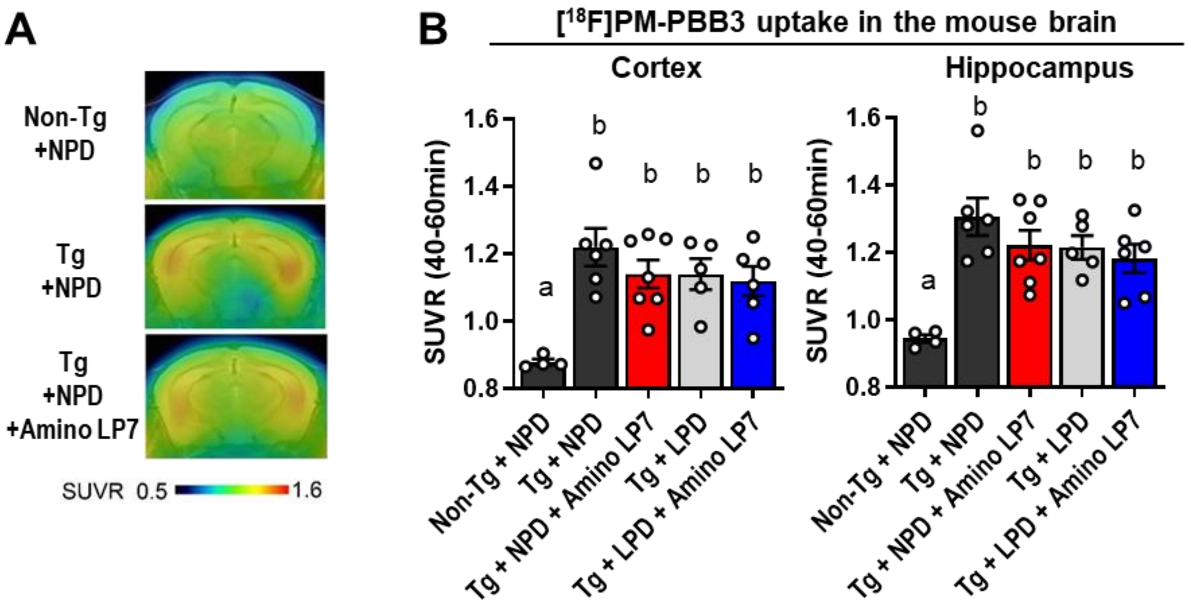

Fig. S2: [^{18}F]PM-PBB3 uptake in rTg4510 mice is not affected by a low protein diet or Amino LP7.

(**A**) Representative PET images of 6.5-month-old nontransgenic and rTg4510 mice at 40–60 min after intravenous administration of [^{18}F]PM-PBB3-PET are shown. PET images are superimposed on individual MRI data and generated using the cerebellum as a reference region. (**B**) Standardized uptake value ratio (SUVR) in the cortex (left) and hippocampus (right) using the cerebellum as a reference region at 40–60 min post-[^{18}F]PM-PBB3 injection in rTg4510 mice under NPD or LPD conditions with or without Amino LP7 administration and their littermate controls under NPD (n = 3 for each condition). ANOVA (cortex, $F_{(4,23)} = 6.40$, $P = 0.001$; hippocampus, $F_{(4,23)} = 7.47$, $P = 0.0005$) with Tukey's post hoc test was used. All data are expressed as the mean ± SEM. Points represent individual animals. The difference between the means is not statistically significant ($P \geq 0.05$) for all groups with the same alphabetical symbols and is statistically significant ($P < 0.05$) for two groups with different symbols. Non-Tg, littermate control; Tg, rTg4510.

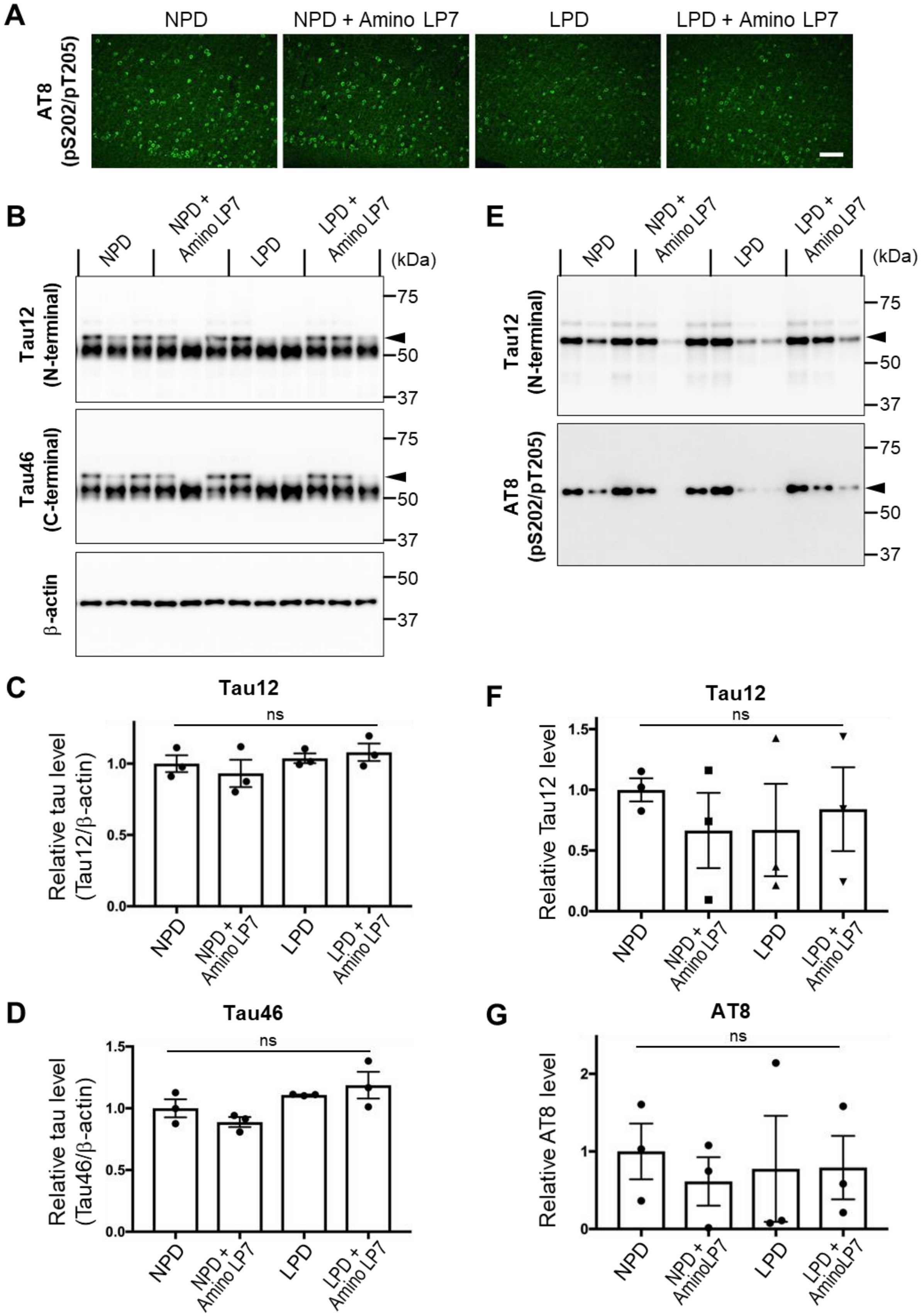
A
NPD
NPD + Amino LP7
LPD
LPD + Amino LP7
AT8 (pS202/pT205)
B
NPD
NPD + Amino LP7
LPD
LPD + Amino LP7
(kDa)
Tau12 (N-terminal)
Tau46 (C-terminal)
β-actin
75
50
37
C
Tau12
ns
Relative tau level (Tau12/β-actin)
1.0
0.5
0.0
D
Tau46
ns
Relative tau level (Tau46/β-actin)
1.5
1.0
0.5
0.0
E
NPD
NPD + Amino LP7
LPD
LPD + Amino LP7
(kDa)
Tau12 (N-terminal)
AT8 (pS202/pT205)
F
Tau12
ns
Relative Tau12 level
1.5
1.0
0.5
0.0
G
AT8
ns
Relative AT8 level
2
1
0
NPD
NPD + AminoLP7
LPD
LPD + AminoLP7

Fig. S3: Biochemical analysis of tau protein in 6-month-old rTg4510 mice.
Mice were treated with NPD, NPD + Amino LP7, LPD, or LPD + Amino LP7 for 3 months. (**A**) Immunostaining of cortical sections from rTg4510 mice with AT8 antibodies. Scale bars, 100 μm. (**B**) Western blots for detecting TBS-extractable tau in rTg4510 mice. Samples from the S1 fraction (loading sample containing 0.025 mg wet-weight of brain) were separated by SDS-PAGE, and then Western blotting with Tau12, Tau46 and β-actin antibodies was conducted. Arrowheads indicate hyperphosphorylated 64 kDa tau. (**C** and **D**) Quantitative analysis of tau protein level. Signal intensities of 50–60 kDa bands labeled by Tau12 (C) and Tau46 (D) antibodies were normalized by β-actin signal intensities. There was no significant difference among the four groups by one-way ANOVA with multiple comparison analysis. (**E**) Western blots for detecting sarkosyl-insoluble tau in rTg4510 mice. Samples from the P3 fraction (loading sample containing 0.5 mg wet-weight of brain) were separated by SDS-PAGE, and then Western blotting with Tau12 and AT8 antibodies was conducted. Arrowheads indicate hyperphosphorylated 64 kDa tau. (**F** and **G**) Quantitative analysis of tau protein level. Signal intensities of the 64 kDa band labeled by Tau12 (F) and AT8 (G) antibodies were compared among the four groups. Values are mean ± SEM. Averaged signal in NPD group was set as one. There was no significant difference among the four groups by one-way ANOVA with multiple comparison analysis. ns, not significant.

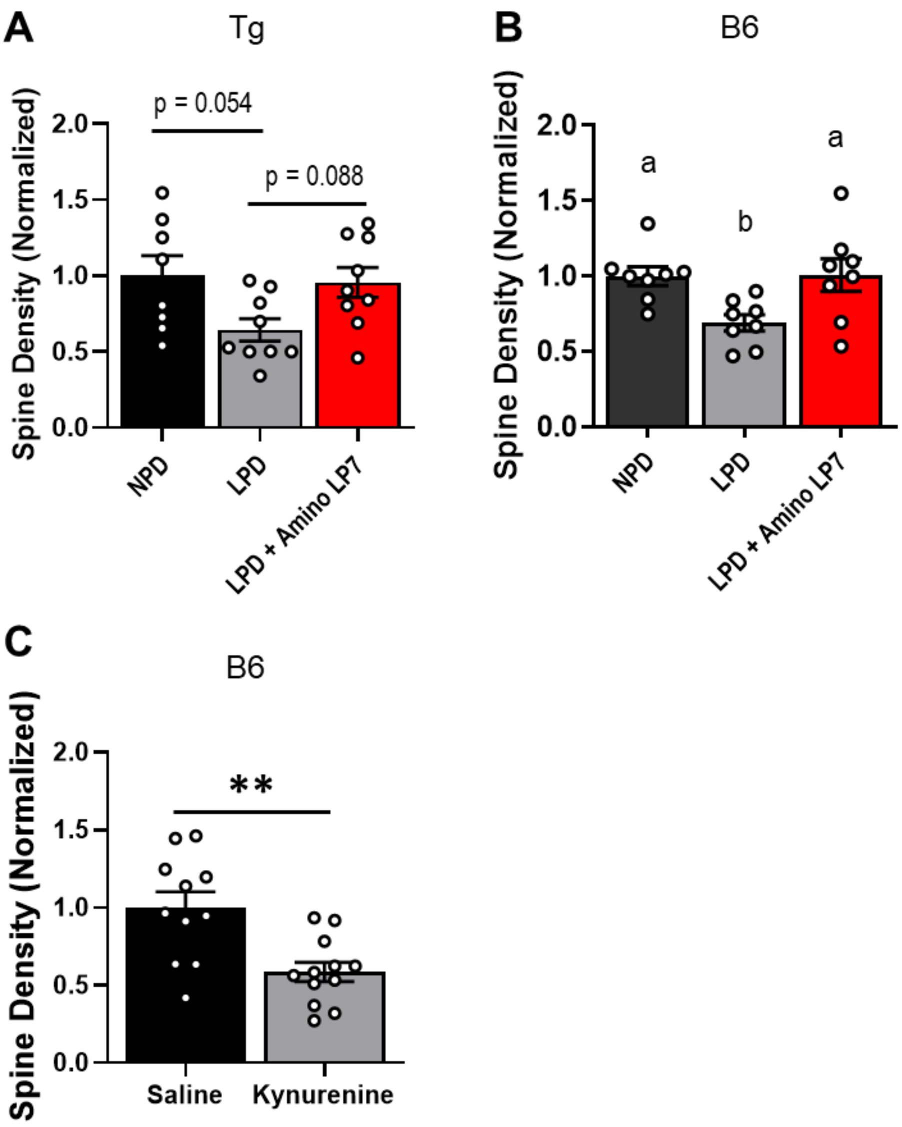

Fig. S4: Spine density is changed in each condition.
(**A** and **B**) Mean spine density in the sensory cortex of rTg4510 mice (A) and aged C57BL/6J mice (B) under NPD, LPD, or LPD with Amino LP7 administration (*n* = 8-9 dendrites from 4 animals in each group). ANOVA (rTg4510, $F_{(2,23)} = 3.74$, $P = 0.039$; aged C57BL/6J, $F_{(2,21)} = 5.28$, $P = 0.013$) with Tukey's post hoc test was used. (**C**) Decreased spine density in the sensory cortex of aged C57BL/6J mice injected with kynurenine compared with the saline group (saline, $n = 11$ dendrites from 4 animals; kynurenine, $n = 12$ dendrites from 4 animals, **: $P < 0.01$ unpaired *t*-test). All data are expressed as the mean ± SEM. Points represent individual dendrites. The difference between the means is not statistically significant ($P \geq 0.05$) for all groups with the same alphabetical symbols and is statistically significant ($P < 0.05$) for two groups with different symbols. Tg, rTg4510; B6, aged C57BL/6J.

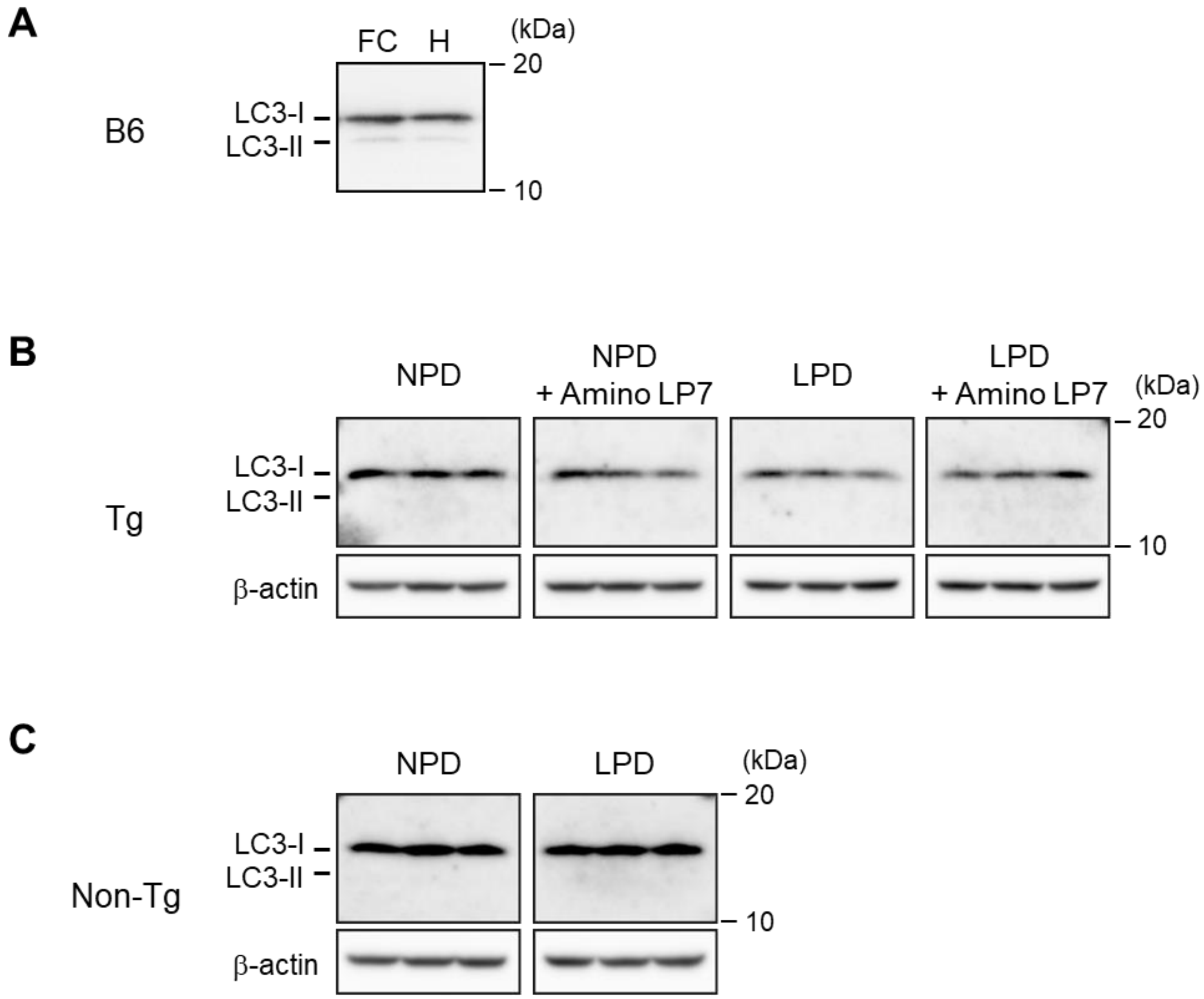

Fig. S5: The level of autophagosome formation in rTg4510 mice is not affected by a low protein diet or Amino LP7.
(**A**) Two microliters of total homogenates from the frontal cortex (FC) and hippocampus (H) of 9-month-old C57BL/6J mice were run on SDS-PAGE and immunoblotted with anti-LC3 antibody. LC3-II, serving as an indicator of autophagosome formation because the total amount is closely correlated with the number of autophagosomes, was detected in the frontal cortex and hippocampus of C57BL/6J mice. (**B** and **C**) Two microliters of total homogenates from the forebrain of 6.5-month-old rTg4510 mice under NPD or LPD with or without Amino LP7 administration (B, $n = 3$ for each condition) and nontransgenic mice under NPD or LPD (C, $n = 3$ for each condition) were run on SDS-PAGE and immunoblotted with anti-LC3 and anti-β-actin antibodies. LC3-II was not clearly detected in the forebrain of all groups, suggesting that there was no clear difference in the level of autophagosome formation between the groups. Non-Tg, littermate control; Tg, rTg4510; B6, C57BL/6J.

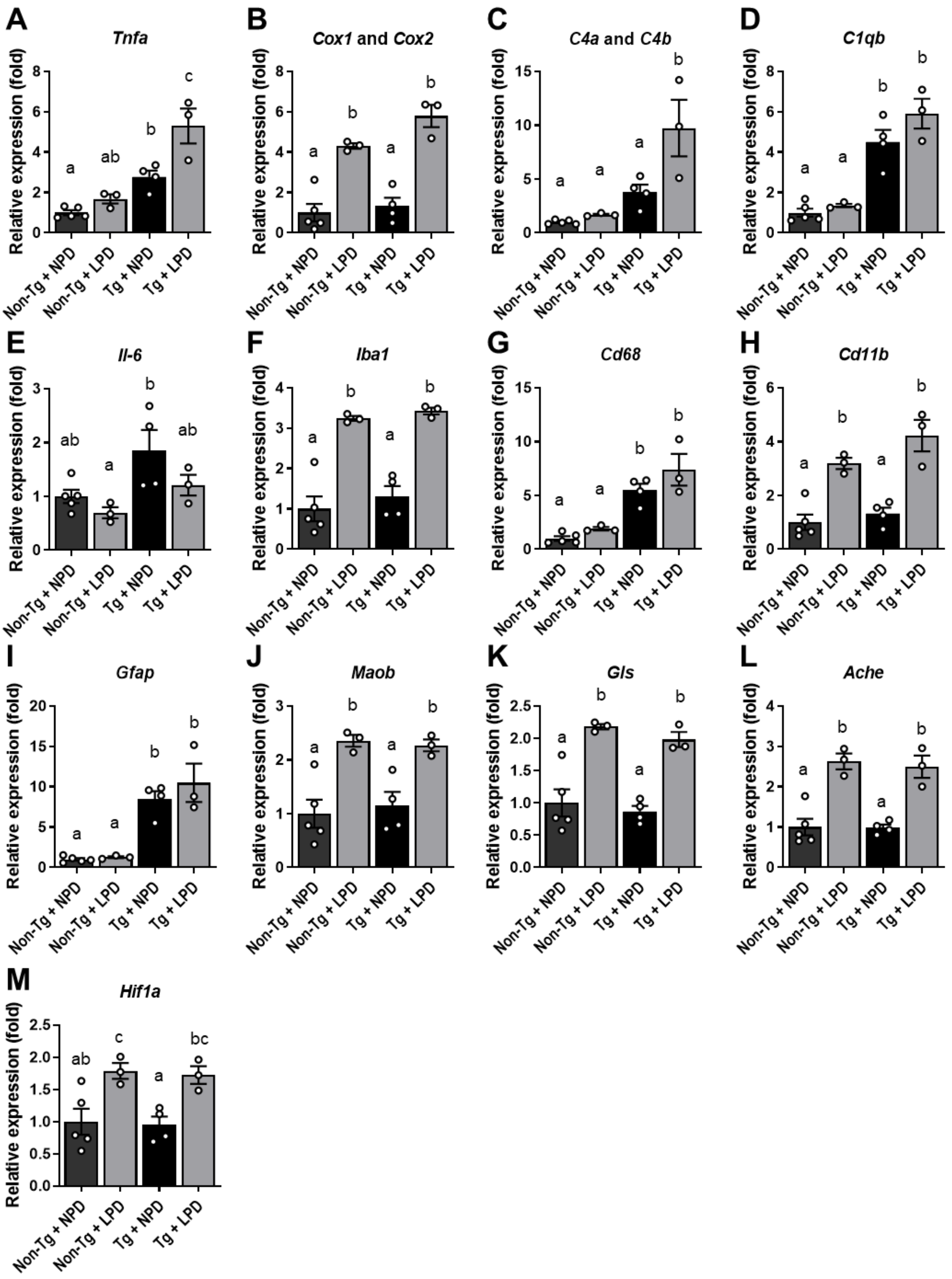
A
Tnfa
B
Cox1 and Cox2
C
C4a and C4b
D
C1qb
E
Il-6
F
Iba1
G
Cd68
H
Cd11b
I
Gfap
J
Maob
K
Gls
L
Ache
M
Hif1a
Relative expression (fold)
Non-Tg + NPD
Non-Tg + LPD
Tg + NPD
Tg + LPD
a
ab
b
c
bc

Fig. S6: Quantification of gene expression in rTg4510 mice

Expression levels in the cerebral cortex of *Tnfa* (**A**), *Cox1* and *Cox2* (**B**), *C4a* and *C4b* (**C**), *C1qb* (**D**), *Il-6* (**E**), *Iba-1* (**F**), *Cd68* (**G**), *Cd11b* (**H**), *Gfap* (**I**), *Maob* (**J**), *Gls* (**K**), *Ache* (**L**), and *Hif1a* (**M**) in rTg4510 mice in each food condition. The expression levels of each gene were determined by RT-qPCR relative to the housekeeping gene *Gapdh*. ANOVA (*Tnfa*, $F_{(3,11)} = 21.59$, $P < 0.0001$; *Cox1* and *Cox2*, $F_{(3,11)} = 27.78$, $P < 0.0001$; *C4a* and *C4b*, $F_{(3,11)} = 12.24$, $P = 0.001$; *C1qb*, $F_{(3,11)} = 27.07$, $P < 0.0001$; *Il-6*, $F_{(3,11)} = 4.32$, $P = 0.031$; *Iba1*, $F_{(3,11)} = 23.57$, $P < 0.0001$; *Cd68*, $F_{(3,11)} = 19.94$, $P < 0.0001$; *Cd11b*, $F_{(3,11)} = 20.51$, $P < 0.0001$; *Gfap*, $F_{(3,11)} = 20.42$, $P < 0.0001$; *Maob*, $F_{(3,11)} = 9.11$, $P = 0.003$; *Gls*, $F_{(3,11)} = 16.52$, $P = 0.0002$; *Ache*, $F_{(3,11)} = 20.34$, $P < 0.0001$; *Hif1a*, $F_{(3,11)} = 6.66$, $P = 0.008$) with Tukey's post hoc test was used. All data are expressed as the mean ± SEM. Points represent individual animals. The difference between the means is not statistically significant ($P \geq 0.05$) for all groups with the same alphabetical symbols and is statistically significant ($P < 0.05$) for two groups with different symbols. Non-Tg, littermate control; Tg, rTg4510.

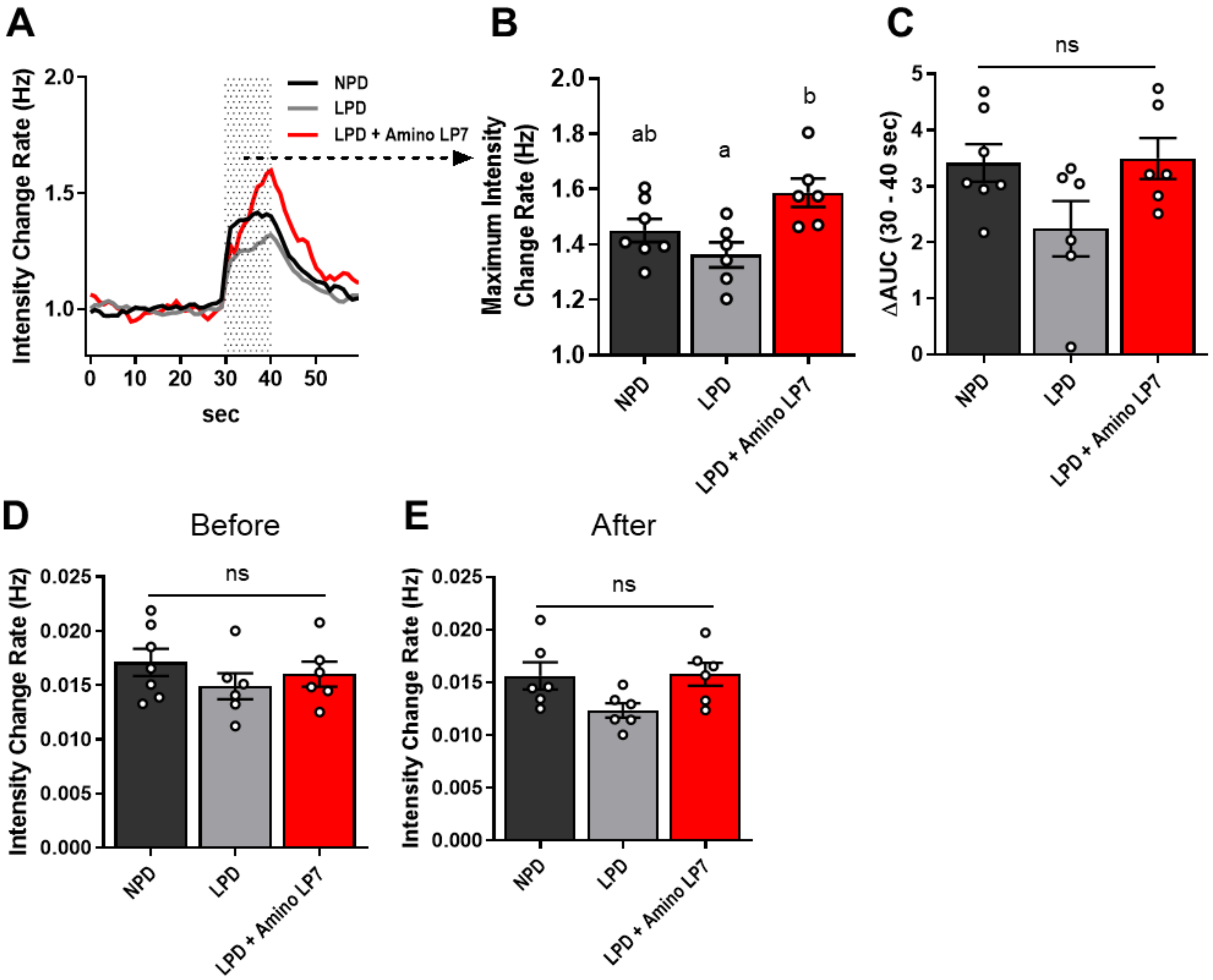

Fig. S7: Evoked synaptic responses are affected by a low protein diet and Amino LP7 in aged C57BL/6J mice.

(**A** to **C**) Time course (A), mean maximum intensity change rate (B) and mean ΔAUC intensity change rate (C) evoked by 10 s whisker stimulation in the barrel cortex of aged C57BL/6J mice under NPD, LPD, or LPD with Amino LP7 administration (*n* = 6-7 animals in each group). ANOVA (maximum intensity change rate, $F_{(2,16)} = 5.75$, $P = 0.013$; ΔAUC intensity change rate, $F_{(2,16)} = 3.00$, $P = 0.078$) with Tukey's post hoc test was used. (**D** and **E**) Mean spontaneous intensity change rate during 240 s before (D) and after (E) experimental diet intake in each group. All data are expressed as the mean ± SEM. Points represent individual animals. The difference between the means is not statistically significant ($P \geq 0.05$) for all groups with the same alphabetical symbols and is statistically significant ($P < 0.05$) for two groups with different symbols. ns, not significant.

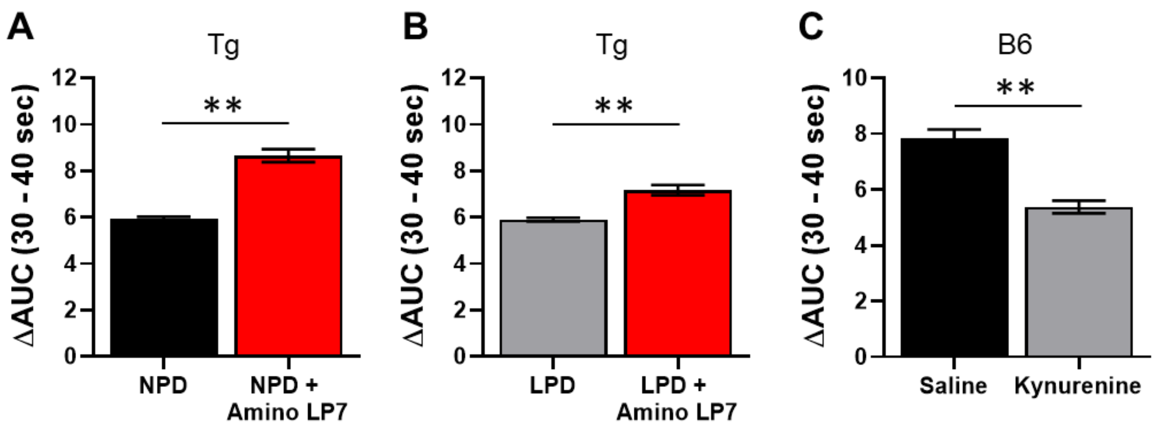

Fig. S8: Evoked synaptic responses are affected by a low protein diet and Amino LP7 in rTg4510 mice and by kynurenine in aged C57BL/6J mice.
(**A**) Mean ΔAUC intensity change rate in rTg4510 mice under NPD with or without Amino LP7 administration (n = 436–1655 cells from 3 animals in each group). (**B**) Mean ΔAUC intensity change rate in rTg4510 mice under LPD with or without Amino LP7 administration (n = 769–718 cells from 2 animals in each group). (**C**) Mean ΔAUC intensity change rate in B6 mice under NPD with saline or Amino LP7 administration (n = 533–624 cells from 4 animals in each group). All data are expressed as the mean ± SEM. **: $P < 0.01$, unpaired t-test. Tg, rTg4510; B6, C57BL/6J.

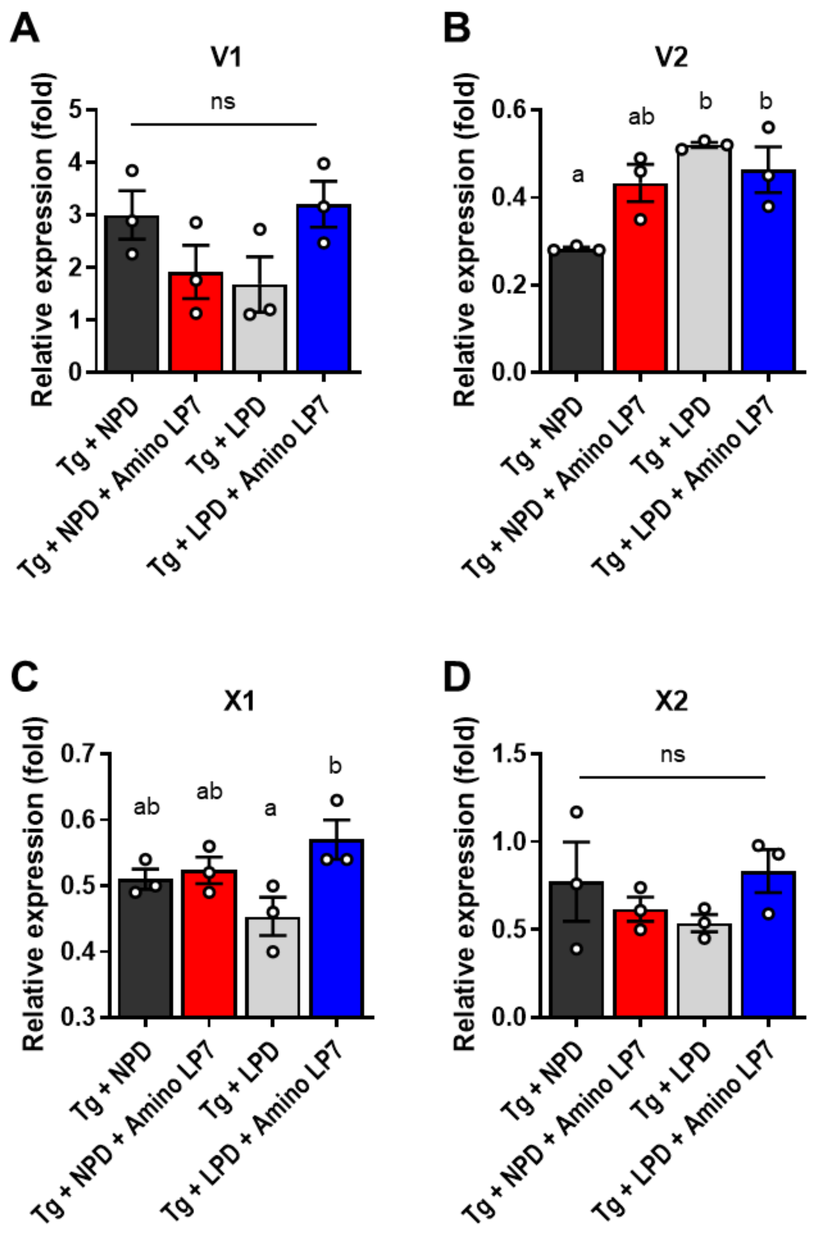

Fig. S9: Expression levels of *Fgf14* variants V1, V2, X1 and X2 in rTg4510 mice.
Expression levels in the cerebral cortex of *Fgf14* variants V1 (**A**), V2 (**B**), X1 (**C**), and X2 (**D**) in rTg4510 mice in each food condition. The expression levels of each variant were determined by RT-qPCR relative to the housekeeping gene *Hprt*. ANOVA (V2, $F_{(3,8)} = 8.88$, $P = 0.006$; X1, $F_{(3,8)} = 3.86$, $P = 0.056$) with Tukey's post hoc test was used. The expression levels of the *Fgf14* variants V1, V2 and X1 were significantly different between NPD-fed WT and rTg4510 mice (*40*). All data were normalized to NPD-fed WT mice. All data are expressed as the mean ± SEM. Points represent individual animals. The difference between the means is not statistically significant ($P \geq 0.05$) for all groups with the same alphabetical symbols and is statistically significant ($P < 0.05$) for two groups with different symbols. ns, not significant; Tg, rTg4510.

Table S1: Composition of experimental diets

	NPD (%)	LPD (%)
Casein	20.00	5.00
Cornstarch	39.75	51.01
α-Starch	13.20	16.94
Sucrose	10.00	10.00
Soybean oil	7.00	7.00
Cellulose	5.00	5.00
Vitamin mix (AIN93)	1.00	1.00
Mineral mix (AIN93G)	3.50	3.50
Cystine	0.30	0.30
Choline bitartrate	0.25	0.25
TBHQ	0.0014	0.0014
Total	100	100

TBHQ, tertiary butylhydroquinone.

Table S2: Comparison of amino acid composition of casein and Amino LP7

%, w/w	Casein composition	Amino LP7
Alanine	2.80	-
Arginine	3.61	-
Aspartic acid	6.59	-
Cystine	0.27	-
Glutamate	20.76	-
Glycine	1.90	-
Histidine	2.62	5.4
Isoleucine	5.14	8.6
Leucine	9.39	31.1
Lysine	7.49	22.1
Methionine	2.53	-
Phenylalanine	4.60	28.3
Proline	10.11	-
Serine	5.23	-
Threonine	4.15	-
Tryptophan	1.26	0.7
Tyrosine	5.42	-
Valine	6.14	3.9
Total	100	100

Table S3: Plasma concentrations of essential amino acids

Amino Acids (μM), mean ± SEM.

	Non-Tg (n = 7)	Tg + NPD (n = 6)	Tg + NPD + Amino LP7 (n = 9)	Tg + LPD (n = 5)	Tg + LPD + Amino LP7 (n = 4)
Leu	167 ± 10.3[ab]	126.7 ± 6.9[ab]	231.6 ± 32.2[a]	92.5 ± 9.4[b]	161 ± 46.2[ab]
Phe	72 ± 2.6[ab]	63.8 ± 6.3[ab]	108.1 ± 15.5[a]	38.7 ± 3.6[b]	89.7 ± 21.1[ab]
Lys	298.3 ± 12.4[a]	379 ± 45.9[a]	679.2 ± 65.0[b]	416.2 ± 28.8[ac]	723.8 ± 115.3[bc]
His	77.9 ± 3.7	74.1 ± 1.3	85 ± 2.2	75.4 ± 4.1	93.3 ± 8.9
Ile	98.3 ± 5.8[a]	74.3 ± 5.3[ab]	94.3 ± 9.0[a]	51.5 ± 5.8[b]	53.9 ± 11.5[b]
Val	248.6 ± 10.4[a]	193.2 ± 17.6[ab]	220.6 ± 20.2[ab]	166.2 ± 13.2[bc]	105.8 ± 6.0[c]
Trp	91.4 ± 4.0[a]	110.6 ± 6.0[a]	105.4 ± 6.1[a]	85.7 ± 4.7[ab]	59.8 ± 3.7[b]
Met	113.1 ± 7.8[a]	58.1 ± 5.7[b]	65.1 ± 5.6[b]	60.8 ± 4.5[b]	35.6 ± 4.1[b]
Tyr	72.7 ± 5.8[a]	88.1 ± 11.4[ab]	227.2 ± 27.8[c]	73.7 ± 5.8[a]	224.5 ± 56.9[bc]
Thr	191 ± 13.4[ab]	201 ± 18.7[a]	238.8 ± 16.4[a]	180 ± 14.0[ab]	117.7 ± 10.4[b]
Total	1357.6 ± 63.7[a]	1280.7 ± 58.1[a]	1828 ± 124.0[b]	1167.1 ± 76.8[a]	1440.3 ± 208.4[ab]

The difference between the means is not statistically significant ($P \geq 0.05$) for all groups with the same alphabetical symbols and is statistically significant ($P < 0.05$) for two groups with different symbols. Non-Tg, littermate control; Tg, rTg4510.

Table S4: Primer sequences used for relative RT-qPCR

Target	Primers	
	Forward	Reverse
Tnfa	5’- ATGGCCTCCCTCTCATCAGT -3’	5’- TTTGCTACGACGTGGGCTAC -3’
C1qb	5’- AAGGTGCCTGGCCTCTACTA -3’	5’- TTTCTGCATGCTGTCCCGAT -3’
Cox1 and *Cox2*	5’- ATGAGTCGAAGGAGTCTCTCG -3’	5’- GCACGGATAGTAACAACAGGGA -3’
C4a and *C4b*	5’- TGGTGTGAGCCTGTCCAAAA -3’	5’- AACACGAGTTGGCTTGGCTA -3’
Il-6	5’- TAGTCCTTCCTACCCCAATTTCC -3’	5’- TTGGTCCTTAGCCACTCCTTC -3’
Iba-1	5’- TCCTCCGGCCCATGATTAAAG -3’	5’- CTGTCTGGCTGCCCATTCT -3’
Cd68	5’- TGTCTGATCTTGCTAGGACCG -3’	5’- GAGAGTAACGGCCTTTTTGTGA -3’
Cd11b	5’- ATGGACGCTGATGGCAATACC -3’	5’- TCCCCATTCACGTCTCCCA -3’
Gfap	5’- GGGGCAAAAGCACCAAAGAAG -3’	5’- GGGACAACTTGTATTGTGAGCC -3’
Maob	5’- ATGAGCAACAAAAGCGATGTGA -3’	5’- TCCTAATTGTGTAAGTCCTGCCT -3’
Gls	5’- CTGTTTGCCGCATACACTGG -3’	5’- TAATCCCGCTGCTCCATGTC -3’
Ache	5’- TGGTCACCATGCAGAGGATCT -3’	5’- AGGTAGCGGTTGAACTTCTCC -3’
Hif1a	5’- GGGGAGGACGATGAACATCAA -3’	5’- GGGTGGTTTCTTGTACCCACA -3’
Gapdh	5’- GGGGCAAAAGCACCAAAGAAG -3’	5’- GGGACAACTTGTATTGTGAGCC -3’
Fgf14 variant V1	5’- CATCTTCGGCCTCAAGAAGC -3’	5’- TCAGAACACCTGAGGATCTGGC -3’
Fgf14 variant V2	5’- GGCCTCTTCTTTCTCAGGGT -3’	5’- CATGCAAGGATGGTTCTCGG -3’
Fgf14 variant X1	5’- TGCTAGGCTGAGCAATGTAG -3’	5’- CCTTGGATCTTCGATAGGGC -3’
Fgf14 variant X2	5’- ATGCTGCAGTGTCTTTGTGG -3’	5’- TCACAGGGGATGCTCAGAAG -3’
Hprt	5’- GCTGGTGAAAAGGACCTCT -3’	5’- CCACAGGACTAGAACACCTGCTA -3’
